# Amplitude of low-frequency fluctuation after a single-trigger pain in patients with classical trigeminal neuralgia

**DOI:** 10.1186/s10194-022-01488-8

**Published:** 2022-09-08

**Authors:** Xiuhong Ge, Luoyu Wang, Lei Pan, Haiqi Ye, Xiaofen Zhu, Sandra Fan, Qi Feng, Wenhua Yu, Zhongxiang Ding

**Affiliations:** 1 Department of Radiology, Hangzhou First People’s Hospital, Zhejiang University School of Medicine, Hangzhou, 310000 P.R. China; 2 Department of Radiology, Key Laboratory of Clinical Cancer Pharmacology and Toxicology Research of Zhejiang Province, Affiliated Hangzhou First People’s Hospital, Cancer Center, Zhejiang University School of Medicine, Hangzhou City, 310006 China; 3 Zhejiang Chinese Medical University, Hangzhou, 310000 P.R. China; 4 Department of Neurosurgery, Hangzhou First People’s Hospital, Zhejiang University School of Medicine, No.261, Huansha Road, Shangcheng Distric, Hangzhou, 310000 P.R. China

**Keywords:** Amplitude of low-frequency fluctuation, Central mechanism, Classical trigeminal neuralgia, Pathophysiology, Resting-state magnetic resonance imaging

## Abstract

**Objective:**

This study aimed to explore the central mechanism of classical trigeminal neuralgia (CTN) by analyzing the static amplitude of low-frequency fluctuation (sALFF) and dynamic amplitude of low-frequency fluctuation (dALFF) in patients with CTN before and after a single-trigger pain.

**Methods:**

This study included 48 patients (37 women and 11 men, age 55.65 ± 11.41 years) with CTN. All participants underwent 3D-T1WI and three times resting-state functional magnetic resonance imaging. The images were taken before stimulating the trigger zone (baseline), within 5 s after stimulating the trigger zone (triggering-5 s), and in the 30th minute after stimulating the trigger zone (triggering-30 min). The differences between the three measurements were analyzed using a repeated-measures analysis of variance.

**Results:**

The sALFF values of the bilateral middle occipital gyrus and right cuneus gradually increased, and the values of the left posterior cingulum gyrus and bilateral superior frontal gyrus gradually decreased in triggering-5 s and triggering-30 min. The values of the right middle temporal gyrus and right thalamus decreased in triggering-5 s and subsequently increased in triggering-30 min. The sALFF values of the left superior temporal gyrus increased in triggering-5 s and then decreased in triggering-30 min. The dALFF values of the right fusiform gyrus, bilateral lingual gyrus, left middle temporal gyrus, and right cuneus gyrus gradually increased in both triggering-5 s and triggering-30 min.

**Conclusions:**

The sALFF and dALFF values changed differently in multiple brain regions in triggering-5 s and triggering-30 min of CTN patients after a single trigger of pain, and dALFF is complementary to sALFF. The results might help explore the therapeutic targets for relieving pain and improving the quality of life of patients with CTN.

**Supplementary Information:**

The online version contains supplementary material available at 10.1186/s10194-022-01488-8.

## Introduction

Trigeminal neuralgia (TN) is a severe chronic neurogenic pain, with predominantly right-sided onset [1] and an approximately two to three times higher incidence in women than in men [2, 3]. It is distributed in the trigeminal distribution area, and mostly in the maxillary and mandibular branches. TN is a sudden, severe, transient, and shock-like pain triggered by harmless movements in daily life, often without pain symptoms between pain attacks [4]. TN is known as the most severe pain that humans can withstand [5] and can lead to mental illnesses such as anxiety and depression [1, 6], which can seriously affect patients’ quality of life. According to the etiology of TN, it is classified into three subforms, which are classical TN (CTN), secondary TN (STN) and idiopathic TN (ITN) [7].

Neurovascular compression (NVC) is considered to be the main cause of CTN [8], and demonstration on MRI or during surgery of neurovascular compression (not simply contact), with morphological changes in the trigeminal nerve root [7]. That is, the offending vessel compresses the trigeminal nerve root entry zone, which results in the demyelination and axonal loss of the trigeminal nerve, resulting in a short circuit between pain fibers and non-pain fibers, and thus causing pain [9, 10]. It is currently believed that, besides the NVC, the central nervous system is also involved in the pathogenesis of CTN, which is the central mechanism [4, 9, 11]. The central mechanism manifesting as hyperalgesia and ectopic pain [12] can promote or reduce the involvement of descending inhibitory pathways, which plays an important role in CTN pathogenesis [13]. Therefore, we hypothesized that the brain function of patients with CTN changed after triggering the pain and played an important role in the pathophysiological process.

Neuroimaging has the advantages of being noninvasive and convenient, and the resting-state function magnetic resonance imaging (rs-fMRI) captures the neural activity in the brain of participants at rest, more closely mirroring the physiological state [14]. It has been widely used in the study of pain diseases including TN [4, 6, 9, 11, 14–22].

The static amplitude of low-frequency fluctuation (sALFF) is one of the commonly used data analysis methods for fMRI, it is a reliable and sensitive technique [23]. sALFF is thought to reflect the strength of local neuronal activity [24] and has demonstrated good-to-moderate test–retest reliability [25]. sALFF has already been used in many studies on diseases, including chronic pain and Alzheimer's disease [15, 18, 26]. Cai et al. [15] found that patients with TN had decreased sALFF values in the right dorsolateral prefrontal lobe, bilateral medial prefrontal lobe, and right middle cingulate gyrus after administering painkillers. Wang et al. [18] found increased sALFF values in the bilateral temporal, occipital, and left middle frontal areas and left middle cingulate gyrus, as well as decreased sALFF values in the right inferior temporal gyrus and medial prefrontal cortex in patients with CTN compared with healthy controls (HC). However, the human brain is a complex and dynamic system, and sALFF fails to respond to neural activity and rapidly changing neural interactions [23].

The dynamic ALFF (dALFF) uses the amplitude of low-frequency fluctuation (ALFF) with sliding window approaches [23]. It is a novel metric reflecting the changes in ALFF over time. dALFF describes the temporal fluctuation in energy expenditure based on the oxygen level and reflects the plasticity and flexibility of spontaneous brain activity [27, 28]. DALFF likely captures useful information missed in sALFF, and can therefore provide complementary information [27]. Chen et al. [29] found that the dALFF values of the bilateral anterior insula, bilateral orbitofrontal cortex, bilateral medial prefrontal cortex, bilateral anterior cingulate cortex (ACC), and left middle frontal gyrus cortex were significantly lower in patients with migraine without aura compared with HC. Also, the dALFF values of the ACC negatively correlated with pain intensity. Luo et al. [27] showed that patients with blepharospasm had increased sALFF values in the left primary motor cortex (PMC) compared with the HC, while increased dALFF variance was seen in the right PMC.

In our study, we reconstructed the pain status of patients with CTN having harmless daily activities and performed multi-time rs-fMRI. We hypothesized that sALFF and dALFF in multiple brain regions of patients with CTN changed differently after triggering pain. We used this to locate the brain regions associated with the development of CTN, thus providing a basis for studying the central mechanism of CTN.

## Materials and methods

All the participants provided written informed consent. This prospective study was approved by the local ethics committee of the Hangzhou First People's Hospital, Zhejiang University School of Medicine (IRB# NO.202107002). It was carried out following the Declaration of Helsinki.

### Participants

Eighty-five patients with CTN were recruited prospectively from the Department of Radiology at Hangzhou First People's Hospital, Zhejiang University School of Medicine, between July 2021 and March 2022.

The inclusion criteria for patients with CTN were as follows: 1) patients diagnosed with CTN according to the third edition of the International Classification of Headache Disorders (ICHD-3), and demonstration on MRI of NVC (not simply contact), with morphological changes (atrophy or dislocation) in the trigeminal nerve root [7]; 2) unilateral pain in the distribution of one or more branches [the ophthalmic (V1), the maxillary (V2), and the mandibular (V3)] of the trigeminal nerve; 3) paroxysmal facial pain precipitated by trigger factors (e.g., light touching of the face, opening of the mouth, and so on); 4) conventional magnetic resonance imaging (MRI) T1WI and T2WI examination revealing no evidence of abnormal brain signals; 5) no additional neurological or sensory deficits in all patients; 6) no previous surgical or other invasive procedures for CTN; 7) no contraindications to magnetic resonance scanning; 8) all patients undergoing microvascular decompression, which found that there were NVC and not only contact, and [9] right-handed patients.

The exclusion criteria were as follows: 1) patients with CTN who had undergone surgical treatment before; 2) headaches and other paroxysmal or chronic pain conditions; 3) a family history of headache or other pain in first-degree relatives; 4) other somatic or psychiatric conditions; 5) left handedness, and [6] contraindications to MRI.

### Experimental design

Patients on analgesic medications were asked to discontinue their medications 12 h before their scheduled scanning sessions. Before the MRI scan, a medical history was taken to determine which zone triggered more pain in daily life, and stimulated the trigger zone within 5 s before the second rs-fMRI scan. The trigger zone was stimulated by the doctors, which was gentle touch by long cotton swab [30]. The foam was used for head fixation to ensure that the patient remained head-still during the scan. All participants underwent 3D-T1WI and three times rs-fMRI. The three times rs-fMRI was performed before stimulating the trigger zone (baseline), within 5 s after stimulating the trigger zone (triggering-5 s), and in the 30th minute after stimulating the trigger zone (triggering-30 min). After the scanning, the patients were asked whether the stimulation caused pain and whether they experienced additional pain during the scan.

### Pain evaluation

If the patients experienced the stimulated pain, the pain would be assessed using the Visual Analogue Scale (VAS) after the scan. The researchers guided patients in rating their pain on a scale of 0–10. A higher score indicated greater pain intensity. A rating of “0” represented no pain, and a rating of “10” meant intolerable pain.

### MRI parameters

All patients underwent MRI using a 3.0 T MRI scanner (Siemens, MAGNETOM Verio, Germany) and an eight-channel phased-array head coil. All participants were instructed to close their eyes, stay awake, and breathe quietly until the scan was completed. The 3D damage-gradient echo sequence functional data were collected and used. The parameters were as follows: 176 structural images (repetition time = 1900 ms; echo time = 2.52 ms; thickness, 1 mm; field of view = 256 × 256 mm^2^; voxel size = 1 × 1 × 1 mm^3^; turning angle = 9 degrees), and 240 functional images (repetition time = 2000 ms; echo time = 30 ms; thickness = 3.2 mm; voxel size = 3.44 × 3.44 × 3.20 mm^3^; turning angle = 90 degrees; field of view = 220 × 220 mm^2^; scan time = 8 min). Each scanning process lasted 15 min.

### Image preprocessing

Rs-fMRI data preprocessing was conducted using the Data Processing and Analysis of Brain Imaging (DPABI) and Sales Process Management 12 (SPM12) toolbox based on the MATLAB platform (MathWorks, MA, USA). The preprocessing pipeline included the following steps: (1) discarding the first 10 volumes of each session to ensure that the MRI signal reached a steady state, (2) slice-timing and head motion correction of the remaining images (participants were excluded if their head motion exceeded the 3-mm maximum displacement in the *x*-, *y*-, or *z*-axis or the 3° angular motion in any direction), (3) normalization of the structural images (T1-weighted images) to the Montreal Neurological Institute (MNI) and re-sampling of the resulting data to obtain 3 × 3 × 3 mm^3^ voxel size, (4) spatial smoothing with an isotropic Gaussian kernel of 6-mm full-width at half-maximum, which aimed to reduce the incomplete effect of registration and improve the image signal-to-noise ratio, (5) removal of linear trend of the time course of the blood oxygenation level-dependent (BOLD) signal, and (6) a noise removal process, including the regression of Friston-24 head motion parameters, cerebrospinal fluid signals, and white matter signals. If the head motion exceeded the 3-mm maximum displacement and the rotation exceeded 3^°^ or the framewise displacement (FD) exceeded 0.5, 4 patients were excluded and the remaining 48 patients with CTN were selected for analysis.

### sALFF calculation

After preprocessing the data, the time series of each voxel was first converted into the frequency domain using a fast Fourier transformation. Then, the square root of the power spectrum in each voxel was computed and averaged across the 0.01- to 0.08-Hz domain and taken as the sALFF value. Finally, the sALFF value of each voxel was divided by the global mean sALFF value within the default brain mask for population comparison.

### dALFF calculation

dALFF was calculated by combining ALFF with the sliding window method, which was highly sensitive for detecting changes over time and examining the changes in indicators throughout the brain. The sliding window length is an important parameter in calculating the resting-state dynamics. A shorter window length increased the risk of introducing spurious fluctuations in the observed dALFF, and a longer window length hindered the description of the dynamics of time variability in ALFF. The minimum window length should be larger than 1/f min, where f min was defined as the minimum frequency of the time series [31]. In this way, spurious fluctuations could be excluded. Thus, a window length of 50 TRs (100 s), with a step size of 2 TRs (4 s) as the optimal parameter, was selected [2]. After calculating the ALFF of all voxels in time windows, each participant received multiple window-based ALFF graphs. Then, we calculated the standard deviation (SD) [32] of each voxel for each participant in all window-based ALFF graphs to measure the dynamics. The step size of 5 TRs (10 s) was applied to further validate the results of dALFF with the different step sizes.

### Statistical analysis

In the Data Processing & Analysis of Brain Imaging (DPABI) software, we compared the sALFF and dALFF values of regional brain activity, which were measured three times for patients with TN using repeated-measures analysis of variance (ANOVA) to examine the differences between groups. Based on Gauss random field theory (between voxels: *P* < 0.001, between groups: *P* < 0.001) for multiple comparison correction. The Spearman correlation analysis was used to assess the association between the average sALFF and dALFF values of significant clusters and pain characteristics.

## Results

### Demographic information and clinical characteristics

**Fig. 1:**
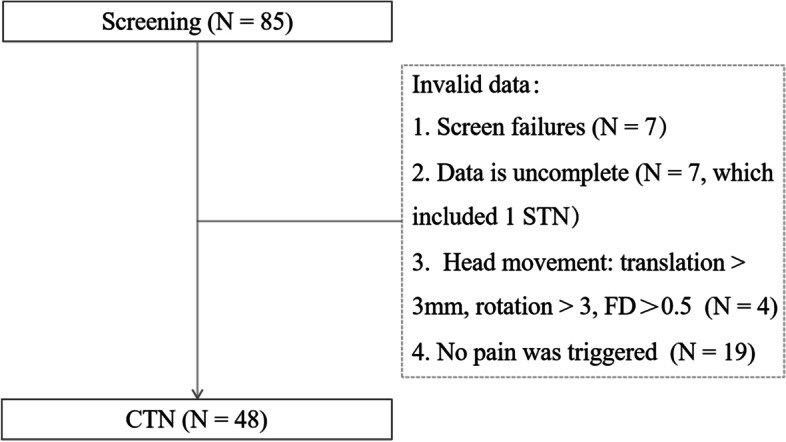
Participant selection. FD, Framewise displacement

A total of 85 patients with CTN participated in the scan, and all them discontinued their medications 12 h before their scheduled scanning sessions and finally 48 patients were included in this study. The procedures for inclusion and exclusion are shown in Fig. 1. The 48 patients with CTN (37 women and 11 men, age 55.65 ± 11.41 years) had a dominant right-sided onset (32/48). The course of the disease, distribution of pain, duration of each pain episode, and pain score are shown in Table 1, and 11 patients with paroxysmal attacks lasting more than 2 min, which may be related to peripheral or central sensitization may account for the continuous pain [7].

**Table 1:** Demographics and behavioral results of CTN

		CTN
Men/Women		11 37
Age (year)		55.65 ± 11.41
Lateral		32R/16L
Duration (year)		3.0 (1.225–6.375)
Average duration of attack (min)	< 1	33
	1 ~ 2	4
	> 2	11
Pain location	V2.3	26
	V3	11
	V2	8
	V1.2	2
	V1.2.3	1
Pain intensity (VAS)		7.85 ± 2.00

*CTN* Classical trigeminal neuralgia, *VAS* Visual Analogue Scale

### sALFF findings after triggering pain in patients with CTN

**Fig. 2:**
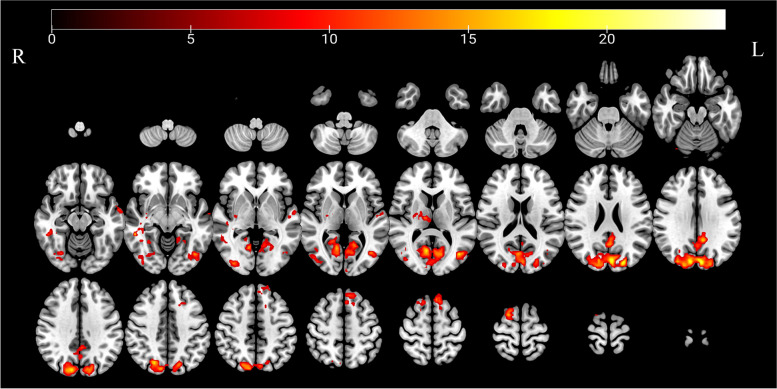
Significant differences in sALFF among different times after triggering the pain in patients with CTN. sALFF, Static amplitude of low-frequency fluctuation; CTN, classical trigeminal neuralgia

**Fig. 3:**
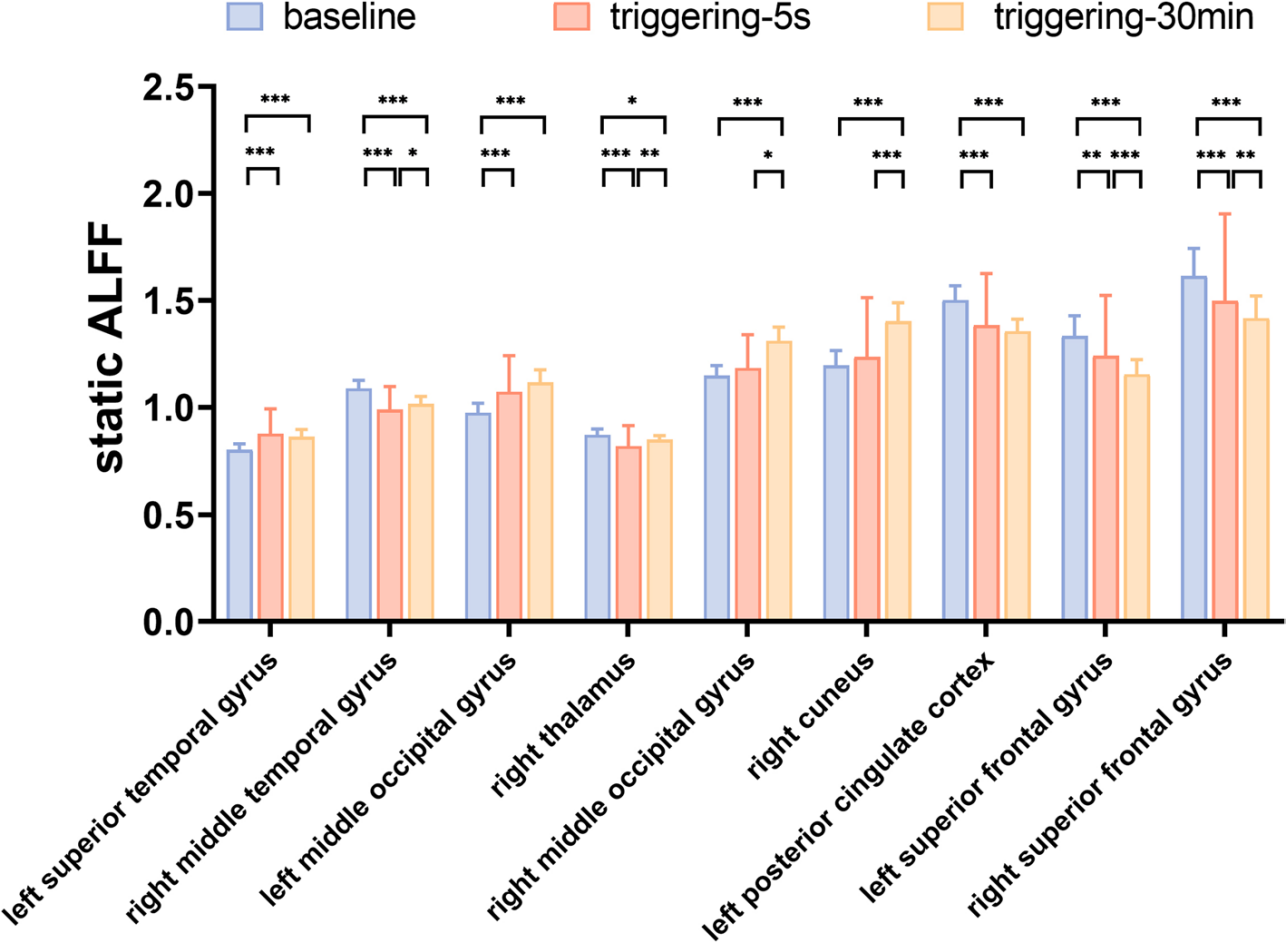
Post hoc comparisons of analysis of variance. The connection between two bars represents significant between-time differences of sALFF (*represents significant level *P* < 0.05, **denotes significant level *P* < 0.01, and *** indicates significant level *P* < 0.001, Bonferroni correction). ALFF, Amplitude of low-frequency fluctuation; sALFF, static amplitude of low-frequency fluctuation; baseline, the rs-fMRI was performed before stimulating the trigger zone; triggering-5s, the rs-fMRI was performed within 5 s after stimulating the trigger zone; triggering-30min, the rs-fMRI was performed in the 30th minute after stimulating the trigger zone

Compared with the baseline, the sALFF values of the bilateral middle occipital gyrus (MOG) and right cuneus gradually increased in triggering-5 s and triggering-30 min, the values for the left posterior cingulum gyrus (PCC) and bilateral superior frontal gyrus (SFG) gradually decreased in triggering-5 s and triggering-30 min, the values of the right middle temporal gyrus (MTG) and right thalamus decreased in triggering-5 s and subsequently increased in triggering-30 min, and the sALFF values of the left superior temporal gyrus (STG) increased in triggering-5 s and then decreased in triggering-30 min (Table 2). The results of the ANOVA and post hoc analysis among the three different times are shown in Figs. 2 and 3.

**Table 2:** Regions with static amplitudes of low-frequency fluctuations in patients with CTN

Brain region	Side	Peak MNI coordinates			Cluster size (voxels)	Peak intensity	*F* value	*P* value	Post hoc* P* value		
		X	Y	Z					Baseline vs 5 s	Baseline vs 30 min	5 s vs 30 min
Superior temporal gyrus	L	–60	–3	–15	51	12.803	40.425	0.000	0.000	0.000	0.915
Middle temporal gyrus	R	42	–33	–6	82	22.348	48.346	0.000	0.000	0.000	0.038
Middle occipital gyrus	L	–42	–72	9	121	23.398	26.863	0.000	0.000	0.000	0.114
Thalamus	R	27	–12	9	59	16.632	20.324	0.000	0.000	0.049	0.003
Middle occipital gyrus	R	36	–87	–3	146	15.357	17.979	0.000	0.179	0.000	0.000
cuneus	R	–6	–84	27	1334	25.222	17.062	0.000	0.550	0.000	0.000
Posterior cingulate cortex	L	–9	–45	27	148	17.867	20.956	0.000	0.000	0.000	0.913
Superior frontal gyrus	L	–6	24	60	137	15.193	15.867	0.000	0.001	0.000	0.000
Superior frontal gyrus	R	18	3	69	72	17.820	25.028	0.000	0.000	0.000	0.004

Baseline, the rs-fMRI was performed before stimulating the trigger zone; 5 s, the rs-fMRI was performed within 5 s after stimulating the trigger zone; 30 min, the rs-fMRI was performed in the 30th minute after stimulating the trigger zone

*CTN* Classical trigeminal neuralgia, *L* Left, *R* Right, *MNI* Montreal Neurological Institute

### The correlation between the average sALFF values of significant brain regions and the pain characteristics

The bilateral SFG and right cuneus were significantly correlated with VAS at baseline, the left PCC was significantly associated with VAS at baseline and triggering-5 s (Table 3).

**Table 3:** The correlation between the different brain regions in sALFF and the VAS, duration among different times after triggering the pain in patients with CTN

Brain region	Side	Duration						VAS					
		baseline		triggering-5 s		triggering-30 min		baseline		triggering-5 s		triggering-30 min	
		*r*	*P*	*r*	*P*	*r*	*P*	*r*	*P*	*r*	*P*	*r*	*P*
Superior temporal gyrus	L	0.266	0.068	0.253	0.083	0.147	0.319	0.106	0.474	0.251	0.085	-0.019	0.898
Middle temporal gyrus	R	-0.281	0.053	-0.134	0.364	-0.099	0.505	0.095	0.520	0.102	0.489	0.204	0.164
Middle occipital gyrus	L	0.246	0.091	0.078	0.600	0.176	0.232	-0.157	0.286	-0.168	0.254	-0.265	0.069
Thalamus	R	-0.065	0.660	-0.142	0.337	-0.225	0.124	-0.243	0.096	-0.216	0.141	-0.227	0.120
Middle occipital gyrus	R	0.159	0.280	0.108	0.464	0.183	0.214	-0.206	0.160	-0.226	0.123	-0.196	0.183
cuneus	R	0.157	0.286	0.001	0.996	0.036	0.808	-0.358	0.013	-0.283	0.051	-0.210	0.152
Posterior cingulate cortex	L	-0.014	0.925	-0.064	0.668	-0.011	0.943	-0.356	0.013	-0.593	0.000	-0.424	0.003
Superior frontal gyrus	L	-0.060	0.686	0.004	0.976	0.031	0.836	0.405	0.004	0.192	0.192	0.231	0.114
Superior frontal gyrus	R	-0.223	0.127	-0.132	0.372	-0.105	0.477	0.320	0.027	0.182	0.216	0.215	0.142

*sALFF* Static amplitude of low-frequency fluctuation, *VAS* Visual Analogue Scale, *CTN* Classical trigeminal neuralgia, *L* Left, *R* Right

### dALFF findings after triggering pain in patients with CTN

**Fig. 4:**
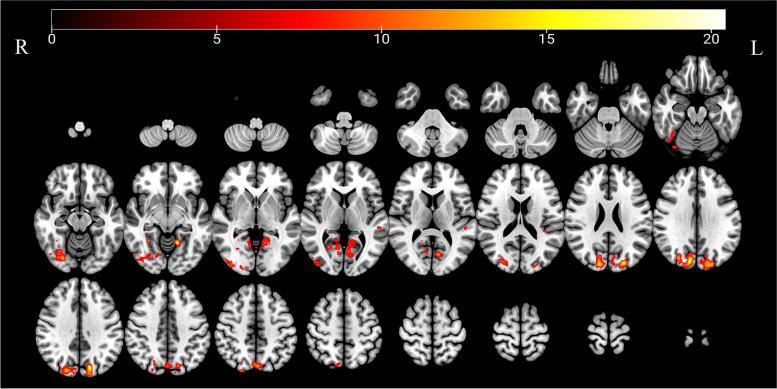
Significant differences in dALFF among different times after triggering the pain in patients with CTN. dALFF, Dynamic amplitude of low-frequency fluctuation; CTN, classical trigeminal neuralgia

**Fig. 5:**
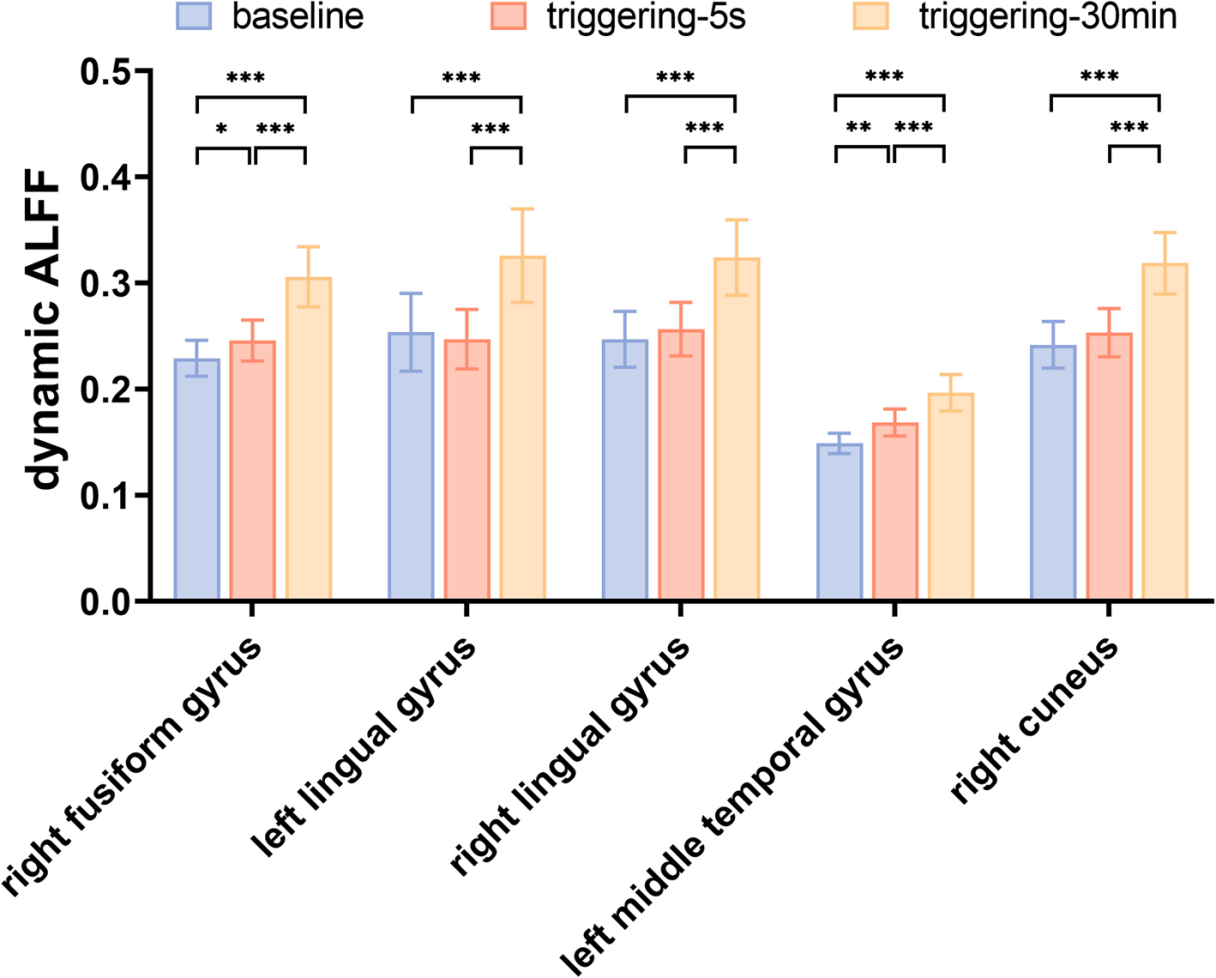
Post hoc comparisons of analysis of variance. The connection between two bars represents significant between-time differences of dALFF (*represents significant level *P* < 0.05, **denotes significant level *P* < 0.01, and ***indicates significant level *P* < 0.001, Bonferroni correction). ALFF, Amplitude of low-frequency fluctuation; dALFF, dynamic amplitude of low-frequency fluctuation; baseline, the rs-fMRI was performed before stimulating the trigger zone; triggering-5s, the rs-fMRI was performed within 5s after stimulating the trigger zone; triggering-30min, the rs-fMRI was performed in the 30th minute after stimulating the trigger zone

Compared with the baseline, the dAFLL values of the right fusiform gyrus, bilateral lingual gyrus, left MTG, and right cuneus gyrus gradually increased in triggering-5 s and triggering-30 min (Table 4). The results of the ANOVA and post hoc analysis among the three different times are shown in Figs. 4 and 5. The results of dALFF with the step size of 5 TRs (10 s) are shown in the supplementary material.

**Table 4:** Regions with dynamical amplitudes of low-frequency fluctuations in patients with CTN

Brain region	Side	Peak MNI coordinates			Cluster size (voxels)	Peak Intensity	*F* value	*P* value	Post hoc* P* value		
		X	Y	Z					Baseline vs 5 s	Baseline vs 30 min	5 s vs 30 min
Fusiform gyrus	R	24	–78	–12	190	15.693	16.151	0.000	0.40	0.000	0.000
Lingual gyrus	L	–15	–54	–9	128	21.027	12.042	0.000	1.000	0.000	0.000
Lingual gyrus	R	30	–51	–9	81	17.524	13.499	0.000	0.967	0.000	0.000
Middle temporal gyrus	L	–54	–30	3	29	11.829	22.405	0.000	0.001	0.000	0.000
Cuneus	R	–21	–87	27	441	20.854	18.351	0.000	0.222	0.000	0.000

Baseline, the rs-fMRI was performed before stimulating the trigger zone; 5 s, the rs-fMRI was performed within 5 s after stimulating the trigger zone; 30 min, the rs-fMRI was performed in the 30th minute after stimulating the trigger zone

*CTN* Classical trigeminal neuralgia, *L* Left, *R* Right, *MNI* Montreal Neurological Institute

### The correlation between the average dALFF values of significant brain regions and the pain characteristics

The right cuneus was significantly correlated with VAS at baseline (Table 5).

**Table 5:** The correlation between the different brain regions in sALFF and the VAS, duration among different times after triggering the pain in patients with CTN

Brain region	Side	Duration						VAS					
		baseline		triggering-5 s		triggering-30 min		baseline		triggering-5 s		triggering-30 min	
		*r*	*P*	*r*	*P*	*r*	*P*	*r*	*P*	*r*	*P*	*r*	*P*
Fusiform gyrus	R	-0.047	0.751	-0.005	0.970	-0.035	0.812	-0.138	0.351	0.025	0.867	0.008	0.956
Lingual gyrus	L	-0.011	0.942	-0.130	0.380	-0.285	0.049	-0.248	0.089	-0.179	0.224	-0.157	0.287
Lingual gyrus	R	-0.036	0.807	-0.107	0.470	-0.255	0.081	-0.257	0.078	-0.046	0.755	-0.041	0.781
Middle temporal gyrus	L	-0.068	0.645	-0.106	0.473	-0.184	0.210	-0.076	0.609	-0.007	0.964	-0.038	0.797
Cuneus	R	-0.029	0.847	-0.087	0.554	-0.130	0.378	-0.323	0.025	-0.209	0.154	-0.064	0.667

*dALFF* Dynamic amplitude of low-frequency fluctuation, *VAS* Visual Analogue Scale, *CTN* Classical trigeminal neuralgia, *L* Left, *R* Right

## Discussion

Our study found that the sALFF and dALFF values changed differently in multiple brain regions during two time points after triggering pain. The sALFF and dALFF values changed in different brain regions, and they also overlapped with each other. The sALFF values demonstrated the steady intensity of activity in brain regions, while the dALFF values reflected the plasticity and flexibility of spontaneous brain activity through the variability of energy expenditure [28]. Both were unique and complementary, providing new insights into the underlying neuropathological mechanisms of the disease. There were several brain regions which the sALFF and dALFF were changed and correlated with VAS.

With the deepening of research on CTN, increasing evidence indicates that the central mechanism is involved in its occurrence and development [4, 30]. Most relevant studies were conducted using rs-fMRI at a single time point [4, 6, 9, 11, 13, 14, 16–22, 33], and a few used fMRI for pain tasks [3, 31, 34, 35]. Although the pain task state could reflect the changes in brain function in patients with TN, the long-term pain was often unbearable and not conducive to universal research, and the pain of CTN was often transient and triggered by harmless movements. In our study, pain episodes in the daily life of patients with CTN were either simulated or reproduced, and the rs-fMRI was collected at multiple time points before and after the pain. It could more accurately reflect the changes in brain function of patients with CTN when the pain attacked.

### Compared with the baseline, the sALFF values increased in triggering-5 s and decreased in triggering-30 min

The STG is located in the temporal lobe. In our study, compared with the baseline, the sALFF values of the left STG increased in triggering-5 s and then decreased in triggering-30 min, but they did not return to the baseline. This finding indicated that the STG not only participated in the process of CTN but also failed to return to the baseline level within half an hour. CTN is a special type of chronic neuropathic pain, and the STG is involved in forming pain memory. Studies have shown that individuals who exaggerate pain show increased activity in the STG during pain stimulation [36]. Mayr et al. [37] found by studying patients with chronic back pain and chronic migraine that STG activity decreased during motor activity, decision-making, and visual input. Bogdanov et al. [38] found by applying sustained thermal stimulation to the participants that the STG activity decreased in patients with migraine compared with HC. This was inconsistent with the results of our study, which might be due to the differences in pain mechanisms in different diseases.

### Compared with the baseline, the sALFF values decreased in triggering-5 s and increased in triggering-30 min

The MTG is part of the default mode network (DMN), which is the most stable resting-state network. The DMN is usually active when it is not in contact with any external environment, and the signal is suppressed when it is subjected to painful stimulation. In our study, the changing pattern of sALFF values in the right MTG during the three different times indicated that it was not only involved in the process of CTN pain but also was part of a trend of recovery in a short time. Yuan et al. [17] found that the signal in the MTG significantly increased in patients with TN compared with HC. A meta-analysis of previous studies on the changes in brain function in patients with CTN found that the changes in the MTG were inconsistent across most studies [39]. It might be because the pain of CTN was paroxysmal and transient. Different studies used different times, and patients had pain during the scanning, resulting in some differences in the study results.

The thalamus is located on both sides of the third ventricle, and the left and right thalamus are connected by a mass of gray matter. This connection participates in the formation of the limbic system and is considered as a "pain matrix". In our study, the signal changes in the thalamus were similar to those in the MTG after triggering the pain, which decreased in triggering-5 s and increased in triggering-30 min. It indicated that the low-frequency oscillations of spontaneous brain activity from the thalamus were interrupted in a short time after triggering pain, and then either recovered or even increased. This further elucidated the important role of the thalamus in the processing of pain. Zhang et al. [20] found by a frequency division study of TN that the ALFF value of the thalamus in the slow-4 band was significantly higher than that in the slow-5 band. The thalamus was also activated in patients with TN during the pain task [32, 34]. In our study, we aimed to reproduce the changes in brain function two times after a single episode of pain in patients with CTN, which could better reflect the pathogenesis of CTN.

### Compared with the baseline, the sALFF values decreased in triggering-5 s and triggering-30 min

Compared with the baseline, the sALFF value in PCC gradually decreased in triggering-5 s and triggering-30 min, with no recovery trend. This indicated that PCC not only participated in the pain process of CTN but also acted for a long time, and the PCC was negatively corrected with VAS at baseline and triggering-5 s. PCC is associated with pain and memory, and it can combine bottom-up attention with information from memory and perception. Borsook et al. [34] found differences in the activation of PCC between spontaneous pain and induced pain in patients with TN; also, a significant decrease in the activation of spontaneous pain was observed. This was broadly in line with our study, which could be due to different participants in this study. The pain of the participants included in our study was induced by harmless actions. In contrast, the pain in the study conducted by Borsook occurred autonomously, and the study had a smaller sample size. Zhang et al. [20] found that the ALFF/fALFF value in PCC decreased in patients with TN in the slow-5 band. The activity of PCC was not only related to pain but more related to the nausea of pain stimulus. The decreased activation of PCC is thought to be part of the integrated reward/disgust circuit [40]. Therefore, we speculated that the ALFF changes of PCC in patients with CTN could be related to pain-induced moods besides triggering pain.

The SFG is located in the upper part of the prefrontal cortex. In this study, the signal change patterns of the bilateral SFG were similar to those of the left PCC, and the SFG was positively corrected with VAS at baseline. The SFG is involved in socially oriented thinking and is also associated with the expectation of impending pain [41]. Mayr et al. [37] found that the activity in SFG decreased both during increased pain and motor activity, decision-making, and visual input in chronic back pain and migraine. This was similar to the results of our study, which indicated that pain triggering in patients with various types of chronic pain could lead to a decrease in the SFG signal. The SFG is associated with cognitive control networks and sensorimotor brain regions [42]. It is an important node in the brain network and working memory [43]. The pain in patients with CTN is triggered by harmless movements in daily life, and patients deliberately restrict movements such as chewing and speaking to avoid pain. Therefore, we speculated that the changes in the SFG signal were not only related to pain but also related to the memory formed by patients' daily restricted movements.

### Compared with the baseline, the sALFF values increased in triggering-5 s and triggering-30 min

Both the MOG and the cuneus are located in the occipital lobe. The occipital lobe contains most of the anatomical areas of the visual cortex, facilitates visual information processing and communication with the cerebral cortex, and plays a role in the perception of facial emotions. Besides visual information processing, it also integrates somatosensory information with other sensory stimuli and cognitive processes, including attention, learning, and memory. In our study, compared with the baseline, the sALFF values in bilateral MOG and right cuneus gradually increased in triggering-5 s and triggering-30 min. Xiang et al. [11] found that the signal in the right MOG increased in patients with CTN when compared with HC. Yuan et al. [17] found that the dALFF of the right cuneus increased in patients with TN compared with HC. Zhang et al. [44] found that patients with herpetic pain experienced changes in cuneus signaling after 6 months of treatment compared with before treatment. Pain often accompanied sensory inputs such as vision, hearing, and smell [13]. In patients with CTN, the signal changes in the MOG and cuneus after pain were related to the changes accompanying vision in addition to pain.

### Compared with the baseline, the dALFF values decreased in triggering-5 s and triggering-30 min

In our study, the sALFF and dALFF signals of the right cuneus changed and gradually increased after triggering pain, which were both negatively corrected with VAS at baseline. No changes were found in sALFF and dALFF values in other brain regions, indicating that dALFF was complementary to sALFF and provided more information for the study of the pathophysiological process of CTN.

Compared with the baseline, the dALFF values of the bilateral lingual gyrus, right fusiform gyrus, and left MTG decreased at different times after stimulating the trigger zone, and the left MTG was positively corrected with average duration of attack at baseline. The lingual gyrus is located in the occipital lobe and below the talate sulcus, which is a part of the primary visual cortex and associated with visual memory and attention deficits. Russo et al. [45] found by thermal stimulation of the trigeminal nerve in the maxillary region that the activity in the higher visual processing areas, including the lingual gyrus, increased in patients with migraine with aura compared with HC and those with migraine without aura. Messina et al. [46] found that the functional connectivity between the right hypothalamus and ipsilateral lingual gyrus decreased in patients with migraine compared with HC, and was associated with a higher frequency of migraine attacks. The lingual gyrus is associated with psychiatric disorders such as depression, anxiety, and panic attacks [47, 48]. The patients with CTN who suffered from pain for a long time were more likely to have anxiety, depression, and other psychiatric disorders. Therefore, in our study, the dALFF values in the bilateral lingual gyrus gradually increased after pain was triggered, which could be related to the pain but also caused by vision or anxiety and depression.

The fusiform gyrus is located on the basal surface of the temporal and occipital lobes, and is involved in sensory integration and cognitive processing. This region is also an important part of the limbic system and is closely associated with mental abilities such as emotion, behavior, learning, and memory. The fusiform gyrus plays an important role in the anticipation and perception of pain regulation [4]. Xiang et al. [11] found that the signal in the right fusiform gyrus increased in patients with CTN compared with HC. Chen et al. [14] found that the ALFF value in the bilateral fusiform gyrus in patients with CTN was significantly higher than that in HC. The results of our study suggested that the right fusiform gyrus played an essential role in the pain process of CTN.

### Limitations

This study had several limitations. Firstly, the central mechanism of CTN was studied by imitating or reproducing the pain state of patients with CTN, but it was only prolonged for 30 min after triggering the pain. The results indicated that it was necessary to prolong the time before scanning so as to better understand the changes in brain function in patients with CTN after an episode of pain. Secondly, we did not conduct the classification studies according to the average duration of attack, and in the future we will study CTN patients depending on the average duration of attack. Thirdly, we only studied the imaging data of patients with CTN before and after pain, and we did not carry out the correlation analysis with clinical parameters.

## Conclusions

Our study was conducted by simulating or reproducing a single pain trigger in patients with CTN. We found that compared with the baseline, the sALFF and dALFF values changed differently in multiple brain regions at different times after a single trigger of pain, and dALFF was complementary to sALFF. The findings provided a basis for exploring the central mechanism of CTN and the therapeutic targets to help patients relieve pain and improve their quality of life.

## Supplementary Information

**Additional file 1: Supplementary file 1.** Regions with dynamical amplitude of low-frequency fluctuations in CTN patients with the step size of 5 TRs (10 s).

## Abbreviations

CTN: Classical trigeminal neuralgia
sALFF: Static amplitude of low-frequency fluctuation
dALFF: Dynamic amplitude of low-frequency fluctuation
ALFF: Amplitude of low-frequency fluctuation
NVC: Neurovascular compression
rs-fMRI: resting-state functional magnetic resonance imaging
HC: Healthy controls
ACC: Anterior cingulate cortex
PMC: Primary motor cortex
ICHD-3: the third edition of the International Classification of Headache Disorders
MRI: Magnetic resonance imaging
VAS: Visual Analogue Scale
SPM12: Sales Process Management 12
DPABI: Data Processing and Analysis of Brain Imaging
MNI: Montreal Neurological Institute
BOLD: Blood oxygenation level-dependent
FD: Framewise displacement
SD: Standard deviation
DPABI: Data Processing & Analysis of Brain Imaging
ANOVA: Analysis of variance
MOG: Middle occipital gyrus
PCC: Posterior cingulum gyrus
SFG: Superior frontal gyrus
MTG: Middle temporal gyrus
STG: Superior temporal gyrus
DMN: Default mode network
