# Supplementary material for: Amplitude of low-frequency fluctuation after a single-trigger pain in patients with classical trigeminal neuralgia
Source: J Headache Pain. 2022 Sep 8;23(1):117. doi: 10.1186/s10194-022-01488-8 (PMC9461270; doi:10.1186/s10194-022-01488-8)
Supplement: Supplementary file 1 — Additional file 1: Supplementary file 1. Regions with dynamical amplitude of low-frequency fluctuations in CTN patients with the step size of 5 TRs (10 s). [file 10194_2022_1488_MOESM1_ESM.docx]

Sup 1. Regions with dynamical amplitude of low-frequency fluctuations in CTN patients with the step size of 5 TRs (10 s).

| Brain region | Side | Peak MNI coordinates | | | Cluster size  (voxels) | Peak Intensity | *F* value | *P* value | Post hoc *P* value | | |
| --- | --- | --- | --- | --- | --- | --- | --- | --- | --- | --- | --- |
|  |  | X | Y | Z |  |  |  |  | Baseline vs 5 s | Baseline vs 30 min | 5 s vs 30 min |
| Fusiform gyrus | R | 24 | -81 | -12 | 69 | 15.9495 | 16.085 | 0.000 | 0.31 | 0.000 | 0.000 |
| Lingual gyrus | L | -15 | -54 | -9 | 65 | 20.9778 | 11.883 | 0.000 | 1.000 | 0.000 | 0.000 |
| Lingual gyrus | R | 30 | -51 | -9 | 38 | 17.4515 | 12.875 | 0.000 | 0.996 | 0.000 | 0.000 |
| Middle temporal gyrus | L | -54 | -30 | 3 | 16 | 11.7673 | 22.789 | 0.000 | 0.000 | 0.000 | 0.000 |
| Cuneus | R | -21 | -87 | 27 | 95 | 21.124 | 18.577 | 0.000 | 0.172 | 0.000 | 0.000 |

CTN, Classical trigeminal neuralgia; L, left; R, right; MNI, Montreal Neurological Institute; baseline, the rs-fMRI was performed before stimulating the trigger zone; 5 s, the rs-fMRI was performed within 5 s after stimulating the trigger zone; 30 min, the rs-fMRI was performed in the 30th minute after stimulating the trigger zone.
